# Social and material deprivation and the cost-effectiveness of an intervention to promote physical activity: cohort study and Markov model

**DOI:** 10.1093/pubmed/fdt132

**Published:** 2014-01-30

**Authors:** Martin Gulliford, Judith Charlton, Nawaraj Bhattarai, Caroline Rudisill

**Affiliations:** 1 Department of Primary Care and Public Health Sciences, King's College London, London, UK; 2 Department of Social Policy, London School of Economics and Political Science, London, UK

**Keywords:** brief intervention, economic evaluation, health inequality, physical activity, primary care, socioeconomic position

## Abstract

**Background:**

We developed a method to model the cost-effectiveness at different levels of deprivation of an intervention to promote physical activity.

**Methods:**

The cost-effectiveness of a brief intervention in primary care was estimated by means of a Markov model stratified by deprivation quintile. Estimates for disease incidence, mortality, depression prevalence and health service utilization were obtained from 282 887 participants in the UK Clinical Practice Research Datalink with linked deprivation scores. Discounted results were compared for least deprived and most deprived quintiles.

**Results:**

An effective intervention to promote physical activity continuing for 5 years gave an increase in life years free from disease: least deprived 54.9 (95% interval 17.5–93.5) per 1000 participants entering model; most deprived 74.5 (22.8–128.0) per 1000. The overall incremental quality adjusted life years were: least deprived, 3.7 per 1000 and most deprived, 6.1 per 1000 with probability cost-effective at £30 000 per QALY being 52.5 and 63.3%, respectively. When the intervention was modelled to be 30% less effective in the most deprived than the least deprived quintile, the probability cost-effective was least deprived 52.9% and most deprived 55.9%.

**Conclusion:**

Physical activity interventions may generate greater health benefits in deprived populations. When intervention effectiveness is attenuated in deprived groups, cost-effectiveness may sometimes still be similar to that in the most affluent groups. Even with favourable assumptions, evidence was insufficient to support wider use of presently available brief primary care interventions in a universal strategy for primary prevention.

## Introduction

Socioeconomic inequalities in health exist in all countries,^1^ with a large body of evidence documenting the extent of these inequalities in developed countries. There are complex, and perhaps poorly understood, pathways linking reduced access to socioeconomic resources to health status.^2^ Injurious exposures arise from the physical environment in which people live, including home, recreational and working conditions; as well as from psychosocial factors, including social relationships, expectations and beliefs.^2^ Material conditions and psychosocial influences may influence individual behaviours, such as smoking, drinking, diet and physical activity, with negative impacts on health status. Lifestyle behaviours have therefore received considerable attention as proximal mediators of the effect of social and material deprivation on health.^3,4^

Research into socioeconomic inequalities in health has progressed through stages of description and analysis and now aims to progress to intervention strategies,^5^ with the objective of tackling inequalities in health through well-defined packages of intervention.^6^ These interventions may be targeted to specific populations or encourage increased overall uptake to reduce disparate outcomes. However, the evidence base to inform appropriate intervention strategies remains under-developed.^6^ Conventional epidemiological designs, including randomized studies at the individual or community level, are generally utilized to evaluate effectiveness as an outcome, rather than to assess how interventions might impact upon measures of inequality.

The present research aimed to develop a modelling approach to facilitate estimation of the cost-effectiveness of interventions at different levels of deprivation. We focus on the promotion of physical activity with the aim of reducing the incidence of potentially disabling long-term conditions. Higher levels of physical activity are associated with lower risk of diabetes mellitus, heart disease, stroke and certain cancers,^7^ but lower socioeconomic groups generally show lower levels of leisure-time physical activity.^8^ A range of interventions has been proposed to promote physical activity.^9^ One approach is through brief interventions delivered in primary care. A systematic review of randomized controlled trials found that brief interventions were associated with higher levels of self-reported physical activity levels at 12 months.^10^ Drawing on this meta-analysis, we modelled the potential long-term health outcomes and cost-effectiveness of an intervention to promote physical activity in primary care. The results from a Markov Model showed that there could be a substantial increase in life-years lived free of physical disease, although there was only weak evidence that intervention could be cost-effective, even when delivered to the general population registered in primary care at very low cost.^11^ The present report extends this research by presenting cost-effectiveness measures estimated at different levels of deprivation. While acknowledging that there was only weak evidence that this type of intervention could be cost-effective, the research aimed to evaluate the extent to which cost-effectiveness might vary across deprivation categories.

The uptake of preventive medical interventions is generally lower in more deprived groups,^12^ reducing their effectiveness. Preventive interventions therefore have the potential to increase inequalities in health.^13^ However, the incidence of disease is higher in lower socioeconomic groups and the potential benefits from intervention are greater here than for more affluent groups. These considerations raise a question concerning the extent to which interventions may be more or less cost-effective in relation to social and material deprivation. The present study aimed to evaluate the impact of deprivation on the cost-effectiveness of an intervention to promote physical activity. The study aimed to determine whether the costs and outcomes of an intervention to promote physical activity were likely to be similar if the intervention was either equally effective at increasing physical activity in different deprivation categories, or less effective in more deprived groups.

## Methods

### Design and purpose

The purpose of the study was to develop an approach to modelling the cost-effectiveness, at different levels of deprivation, of a brief intervention in primary care to promote physical activity. The target population for the study was the general population of adults registered in primary care in the UK. The study intervention was a brief intervention to promote physical activity in healthy individuals registered in primary care. This was considered to have similar effectiveness to that reported by Orrow *et al.*^10^ but required to be delivered at very low cost, equivalent to one general practitioner consultation per person per year. The comparator was ‘usual’ care in which there was no structured approach to promoting physical activity. The primary outcome was evaluated in terms of quality adjusted life years (QALYs), while years lived with morbidity, prevalence of depression and mortality were also evaluated. The model used a lifetime time horizon. Only direct costs to health services were considered.

### Data source and participants

Data for physical activity levels in the English population were drawn from the Health Survey for England (HSE) 2006 and 2008, excluding participants with prevalent disease.^8^ The HSE is an annual household interview survey of the health of the general population in England. The main focus of the survey in 2006 was on antecedents of cardiovascular disease, while in 2008 fitness and physical activity were a major focus. The survey uses a multi-stage cluster sampling design to draw a representative sample of ∼16 000 adults. Data were analysed for a derived summary measure, included in the HSE reports, describing the number of days per week with any physical activity lasting >30 min.^7^ This was reduced to the categories: *inactive*, with less than 1 day per week with physical activity; *insufficient*, with between 1 and 4 days per week with physical activity and *active*, with 5 days of physical activity per week. This is consistent with current recommendations that adults should exercise for at least 30 min on 5 days per week. The proportion of participants in each category was estimated by age-group, sex and quintile of deprivation. Indices of multiple deprivation were used, including the 2004 version for HSE 2006 and the 2007 version for HSE 2008. These incorporate measures of deprivation across domains of income, employment, health and disability, education and training, housing and services, living environment and crime. Age-standardized proportions were estimated using the European Standard Population for reference.

Estimates to inform the model for the incidence and prevalence of disease, mortality, and health-care utilization were obtained from the Clinical Practice Research Datalink (CPRD),^14^ as reported elsewhere.^15,16^ Estimates were obtained from analysis of the electronic health records of 282 887 participants in CPRD for the period 2007–11, using deprivation scores (IMD2010) linked at the individual participant postcode level.^17^ Regression modelling provided estimates to calculate transition probabilities that were incorporated into the model. The incidence of each state, and the mortality in each state, were estimated in a time-to-event framework using case definitions reported previously.^15^ A Weibull model was fitted with the incidence, or mortality, of the state as an outcome and age, gender and deprivation quintile as predictors. The coefficients from the regression model were used to estimate transition probabilities, with uncertainty estimated through Cholesky decomposition of the variance–covariance matrix.^18^ A similar approach was implemented for the estimation of depression prevalence with a logistic regression model. Depression was defined as a clinical diagnosis recorded in year, or a diagnosis ever recorded and anti-depressants prescribed in year. Costs of health-care utilization were estimated using unit cost values from standard reference sources.^19^ Drug prescription costs were calculated by linking each prescription to the drug cost obtained from the First DataBank Europe.^20^ The mean costs by deprivation quintile, age-group, gender and model state were estimated from a two part regression model. At the first stage, a probit model was fitted to estimate the probability of health-care costs being non-zero and at the second stage a general linear model with log link and gamma errors was fitted to model the distribution of positive costs.^15^ The estimated mean value of the predicted costs for each age-group, sex and deprivation quintile, provided the input to the Model.

### Model design and estimation

**Fig. 1:**
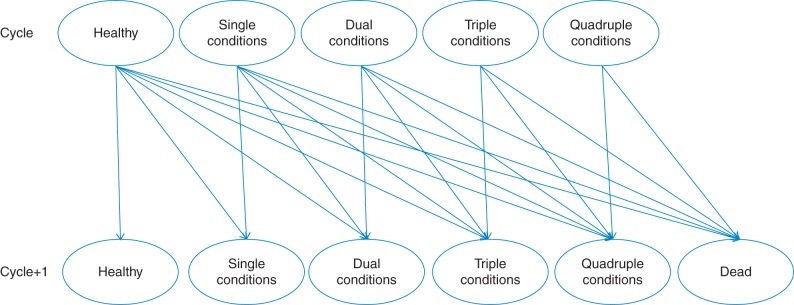
Outline of Markov model. Single conditions: states include diabetes mellitus, coronary heart disease, stroke or colorectal cancer; dual conditions: states include each potential combination of two of these four conditions; triple conditions: states include each potential combination of three of these four conditions; quadruple conditions: state includes all four conditions combined. Individuals in any state may reversibly transition to comorbid depression (not shown) or irreversibly to death. Arrows indicate transitions between states.

A Markov model was designed, as outlined in Fig. 1. A Markov model includes a number of different states, in this case states characterized by the presence of absence of a number of different diseases or death. The cohort within the model is distributed among the states. In our model, all participants entered the model in the healthy state ‘at risk’ but by the end of the Model had either died or reached age 100 and exited the model. Uncertainty in the model is represented in terms of transition probabilities, representing the incidence of disease or mortality; these probabilities determine progression from one state to another. In order to allow estimation of the impact of the intervention, disease incidence, mortality and costs of health-care utilization were evaluated over annual cycles over a lifetime perspective as reported previously.^11^ A Markov model is generally less computationally demanding that an approach requiring individual participant simulation. For these analyses, the Model was run separately for each quintile of deprivation. All Model inputs were stratified by deprivation quintile, in addition to age and sex, and all Model outputs were similarly stratified. Five conditions were included in the Model: diabetes mellitus, coronary heart disease, stroke, colorectal cancer and depression. These conditions were selected because of their association with physical activity.^7^ Individuals without any of these conditions are referred to as ‘at risk’. Individuals at risk enter the model with the same age and sex distribution as that observed in the empirical CPRD population. In each annual cycle of the model, participants may transition to a disease state according to the probabilities estimated from CPRD. Onset of a chronic disease was considered irreversible. Once in a disease state, participants may develop a second, third or fourth disease. Participants in any disease state may progress to death. Each state is also associated with a defined prevalence of depression. There were therefore 17 main states in the Model including, at risk, deceased, and 15 states defined by the potential combinations of the four study conditions; each non-fatal state was further sub-divided into ‘depressed’ and ‘not depressed’ giving a total of 33 states in the Model.

The Markov model was probabilistic and estimated by cohort simulation, implemented through a program written in R software.^21^ After removing participants with prevalent disease, 210 807 healthy participants entered the initial state of the Model, based on the distribution observed in CPRD. All simulations were stratified by single year of age with the initial population agwing by 1 year per cycle. Participants exited the model when they died or reached 100 years of age. The Model was run for each sex separately. Outcomes and costs were compared for Intervention and Standard Care over 70 annual cycles, this allowed the entire cohort to progress either to death or to reach age 100 and exit the model. Utilities for each state were obtained from data published in a compendium of values.^22^

Results were summarized for each deprivation quintile separately. There were 2000 simulations run for each of intervention or standard care scenarios. Results are expressed as rates per 1000 healthy participants entering the model. Mean costs, and the 95% range, were obtained from the data for 2000 simulations. These limits gauge the extent of uncertainty in the results and should be interpreted in terms of the strength of evidence that they provide rather than in terms of statistical significance. Incremental costs and QALYs were obtained as the difference between intervention and standard care scenarios. Costs and QALYs were discounted using a rate of 3.5%, but QALYs were also discounted at a rate of 1.5% as a sensitivity analysis. Net health benefits (NHBs), at a threshold value of £30 000 per QALY as used by the National Institute for Health and Care Excellence, were calculated as the difference between the increment in QALYs and the increment in costs divided by the threshold value of cost per QALY. A cost-effectiveness acceptability curve was plotted using a range of threshold values. The model was implemented with a half-cycle correction for the estimation of QALYs and costs.

### Intervention effects

The intervention was assumed only to modify the incidence of disease in healthy participants at risk. The effect of intervention was estimated using a potential impact fraction (PIF), following Cobiac *et al.*,^23^ as outlined previously.^11^ The PIF provides a means of estimating the extent to which a change in risk factor exposure is associated with a proportionate decline in the likelihood of an individual developing a disease outcome of interest. The PIF was estimated from three sources of data: (i) the effect of brief interventions in primary care on physical activity levels. Orrow *et al.*^10^ estimated that the number needed to treat for an additional sedentary subject to become active was ∼12 with an odds ratio of 1.42 (95% interval 1.17–1.73) and an event rate in control participants of 26% (507/1924); (ii) data for the distribution of physical activity in the general population, by 10-year age group and sex, were obtained from the Health Survey for England as outlined above; (iii) relative risks associating inactivity, or insufficient activity, with the four-study disease outcomes (diabetes, coronary heart disease, stroke and colorectal cancer) were obtained from the World Health Organization study ‘Comparative Quantification of Health Risks'.^7^ The intervention effect was modelled as being maintained for 5 years. The cost of the intervention was modelled as a fixed cost per person year depending on their physical activity level. The population at risk was divided into those that were physically active and those that were physically inactive or who took insufficient physical activity, based on the distribution observed in the Health Survey for England. In the population that was not sufficiently physically active, the intervention cost, in the base case, was modelled as being equivalent to the cost of one family practice consultation per person year (£35).^19^ This could be an entire visit, or a portion of a series of visits, over the course of a year. In the population that was physically active, the cost of screening questions to evaluate physical activity levels was made equivalent to 20% of one family practice consultation per year. Our previous research showed that interventions that are more costly are unlikely to be cost-effective.^11^

### Sensitivity analyses

The probabilistic nature of the model incorporated uncertainty in model inputs directly into the modelling process. Additional sensitivity analyses were implemented to evaluate the impact of varying key assumptions including the rate of discounting; the differential effectiveness of the intervention across deprivation categories; and the impact of differential costs of the intervention across deprivation categories.

## Results

The start population for the Model comprised a population of 210 807 healthy participants drawn from CPRD who were free from morbidity, including 103 267 men and 107 540 women (Table 1). The prevalence of physical inactivity generally showed a graded increase with increasing level of deprivation and this was especially evident in middle age (Table 1). When the categories of ‘inactive’ and ‘insufficiently active’ were combined, inequality by deprivation category was less evident. Values for the relative rates of disease incidence, mortality and depression prevalence are shown in Table 1 for the at risk population and for single-condition states only. In general, there was a graded increase in risk of disease, in mortality and in the prevalence of depression with increasing deprivation category.

**Table 1:** Association of deprivation quintile with disease incidence, mortality and depression prevalence in single condition states.

				*Deprivation quintile*				
	*Data source*	*Units*	*Category*	*Least deprived*	*Second*	*Third*	*Fourth*	*Most deprived*
Population at risk	CPRD	Frequency	Male	23 784	20 109	21 388	18 315	19 671
			Female	25 149	21 171	22 689	18 782	19 749
Mean age (SD)	CPRD	Mean (SD) years	Male	51.1	51.8	51.5	50.4	49.3
			Female	53.1	53.9	53.9	53.2	52.2
Physical inactivity (%)	HSE	Age standardized prevalence	Male	24.2	27.0	28.7	32.4	40.7
			Female	31.9	31.3	33.2	36.5	43.5
Disease incidence	CPRD	Hazard ratio	DM	Reference	1.16	1.29	1.49	1.76
			CHD	Reference	1.10	1.15	1.27	1.47
			Stroke	Reference	1.15	1.26	1.27	1.51
			Colorectal	Reference	1.06	1.05	1.05	1.04
Mortality	CPRD	Hazard ratio	At risk	Reference	1.09	1.16	1.31	1.49
			DM	Reference	0.92	0.95	1.04	1.14
			CHD	Reference	0.98	1.03	1.12	1.19
			Stroke	Reference	1.29	1.12	1.19	1.37
			Colorectal	Reference	0.77	1.06	1.25	1.12
Depression prevalence	CPRD	Odds ratio	At risk	Reference	1.15	1.22	1.44	1.86
			DM	Reference	1.20	1.33	1.43	1.83
			CHD	Reference	1.34	1.33	1.50	2.01
			Stroke	Reference	1.12	1.24	1.28	1.58
			Colorectal	Reference	1.01	0.83	1.36	1.27

Figures derive from coefficients from Weibull models (incidence and mortality) and logistic model (depression prevalence). CHD, coronary heart disease; colorectal, colorectal cancer; CPRD, Clincal Practice Research Datalink; DM, diabetes mellitus type 2; HSE, Health Survey for England.

Table 2 presents results for an intervention in primary care to promote physical activity that continued for 5 years and is assumed to have the same effectiveness regardless of deprivation quintile. Intervention was associated with an increase in life years free from any of the study conditions. The increment was 54.9 per 1000 healthy participants entering the model in the least deprived quintile and 74.5 per 1000 in the most deprived quintile. There was a reduction in life years lived with single morbidities of 43.0 (8.9–78.6) per 1000 in the least deprived quintile and 54.4 (9.0–101.8) in the most deprived quintile, with similar trends in the reduction of dual and triple morbidities. Life years with depression tended to be reduced, even though the intervention had no direct effect on depression, because the prevalence of depression was higher in participants with physical morbidities. Consequently, the reduction tended to be greater in more deprived quintiles.

**Table 2:** Cost-effectiveness of an intervention continued for 5 years with the same intervention effectiveness in each quintile of deprivation.

	*Deprivation quintile*				
	*Least deprived*	*2*	*3*	*4*	*Most deprived*
Number entering intervention	48 933	41 280	44 077	37 097	39 420
Life years lived without disease (per 1000)^a^	54.9 (17.5 to 93.5)	58.7 (17.6 to 99.6)	62.0 (19.5 to 105.9)	69.6 (20.0 to 117.9)	74.5 (22.8 to 128.0)
Life years lived with physical morbidity (per 1000)^a^					
Single condition	−43.0 (−78.6 to −8.9)	−45.1 (−81.9 to −8.1)	−47.7 (−86.6 to −9.5)	−52.5 (−93.9 to −9.6)	−54.4 (−101.8 to −9.0)
Dual conditions	−4.9 (−10.9 to 0.9)	−6.3 (−13.4 to0.95)	−6.2 (−13.4 to 0.6)	−7.5 (−15.8 to 0.95)	−8.9 (−19.4 to 1.60)
Triple conditions	−0.2 (−0.9 to 0.4)	−0.4 (−1.2 to 0.54)	−0.3 (−1.2 to 0.5)	−0.5 (−1.7 to 0.8)	−0.5 (−1.8 to 0.8)
Life years lived with depression (per 1000)^a^	−2.4 (−25.2 to 20.1)	−3.4 (−31.2 to 24.0)	−3.5 (−30.0 to 25.6)	−4.5 (−37.7 to 28.3)	−5.2 (−45.8 to 35.4)
Total intervention costs (£ per 1000)	104 162 (104 144 to 104 179)	105 929 (105 908 to 105 949)	100 962 (100 941 to 100 983)	97 547 (97 524 to 97 570)	102 777 (102 750 to 102 803)
Incremental costs of non-intervention health-care utilization (£ per 1000)	−14 767 (−90 670 to 61 982)	−17 287 (−95 244 to 60 879)	−18 070 (−93 970 to 57 185)	−20 674 (−98 192 to 57 335)	−25 047 (−108 110 to 52 555)
Incremental total costs (£ per 1000)^a^	89 394 (13 483 to 166 160)	88 642 (10 680 to 166 791)	82 892 (7172 to 158 163)	76 873 (−649 to 77 965)	77 730 (−5342 to 155 326)
Incremental QALYs (discounted 3.5%) (per 1000)	3.73 (−10.05 to 18.30)	4.09 (−11.6 to 20.2)	4.45 (−11.7 to 29.7)	4.7 (−12.7 to 21.0)	6.1 (−10.2 to 22.9)
Incremental QALYs (discounted 1.5%) (per 1000)	6.32 (−13.15 to 27.26)	6.9 (−15.8 to 29.2)	7.62 (−15.5 to 29.7)	8.1 (−16.0 to 32.0)	10.3 (−13.7 to 34.8)
Net Health Benefit (QALYs per 1000)	0.75 (−13.32 to 15.38)	1.13 (−14.7 to 17.8)	1.69 (−14.7 to 17.3)	2.11 (−15.7 to 18.8)	3.5 (−13.3 to 20.6)
Probability cost-effective at £30 000 per QALY (%)	52.5	54.0	58.0	58.8	63.3

Figures represent the mean (95% interval) for 2000 simulations, except where indicated.

The discounted costs of delivering the intervention were generally similar across deprivation quintiles (Table 2). However, the reduction in the costs of health-care utilization associated with better health status, and fewer life years lived with physical morbidity or depression, increased with deprivation being −£14 767 per 1 000 in the least deprived quintile and −£25 047 in the most deprived quintile. The overall incremental costs associated with intervention, under this scenario, tended to be lower in the most deprived quintile. The health gain from the intervention, in terms of discounted QALYs, was greatest in the most deprived quintile. When discounted at 3.5%, the gain in QALYs was 3.73 (−10.05 to 18.30) per 1000 in the least deprived Quintile and 6.1 (−10.2 to 22.9) in the most deprived quintile. NHBs were associated with deprivation, being 0.75 QALYs per 1000 in the least deprived quintile and 3.5 QALYs per 1000 in the most deprived quintile. The probability of the intervention being cost-effective at a threshold of £30 000 per QALY was 52.5% in the least deprived quintile and 63.3% in the most deprived quintile.

**Fig. 2:**
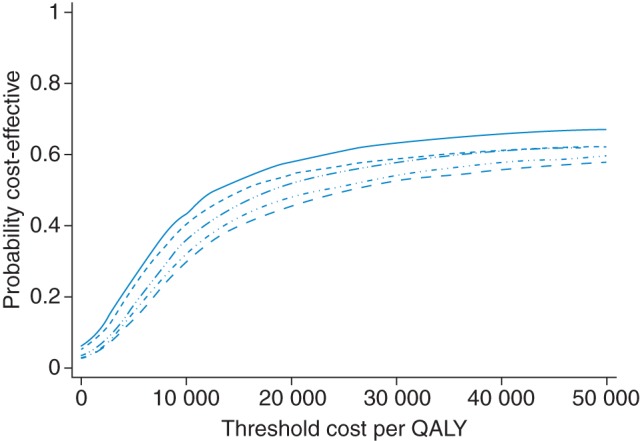
Cost-effectiveness acceptability curves for each quintile of deprivation. Solid line, most deprived quintile; long dashed line, least deprived quintile.

Figure 2 presents cost-effectiveness acceptability curves, plotted for each deprivation quintile, assuming the same level of effectiveness across deprivation quintiles. At any threshold value of cost per QALY, intervention in the most deprived quintile had a substantially higher probability of proving cost-effective than intervention in the least deprived quintile.

In further simulations, we explored the outcomes observed when the intervention was either 20 or 30% less effective in the most deprived quintile when compared with the least deprived. Table 3 shows the distribution of the intervention effects in this scenario. The mean intervention effect of the physical activity intervention on diabetes incidence was 0.966 in the least deprived quintile but 0.974 in the most deprived quintile, when the intervention was modelled to be 20% less effective in this group, and 0.977, when the intervention was modelled to be 30% less effective in the most deprived quintile. In these simulations, the mean NHB was 0.60 (−13.8 to 15.4) QALYs per 1000 in the least deprived quintile, with a probability of 52.9% that the intervention was cost-effective at the threshold of £30 000 per QALY. When the intervention was 20% less effective in the most deprived quintile, compared with the least deprived, the NHBs were 1.93 QALYs per 1000, with a probability of 57.3% that the intervention was cost-effective. When the intervention was modelled as being 30% less effective in the most deprived quintile, NHBs were then similar to those in the least deprived quintile, being 1.60 per 1000, with a probability of 55.9% of the intervention being cost-effective. We also evaluated a scenario in which intervention was twice as costly in the most deprived quintile, with the unit intervention cost equivalent to two GP consultations per year (£70), compared with the least deprived quintile where the unit cost of intervention was equivalent to one GP consultation per year. NHBs in the most deprived quintile were 0.1 (−16.7 to 17.2) QALYs per 1000, probability cost-effective, 50.2%.

**Table 3:** Cost-effectiveness when intervention effect in most deprived quintile is 20 or 30% smaller than in the least deprived quintile.

	*Least deprived, intervention fully effective*		*Most deprived, intervention 20% less effective*		*Most deprived, intervention 30% less effective*	
	*Male*	*Female*	*Male*	*Female*	*Male*	*Female*
Intervention effects (mean, 95% range)						
Diabetes mellitus	0.966 (0.948 to 0.983)	0.967 (0.949 to 0.983)	0.973 (0.960 to 0.987)	0.974 (0.960 to 0.987)	0.977 (0.965 to 0.988)	0.977 (0.966 to 0.988)
Coronary heart disease	0.949 (0.924 to 0.973)	0.950 (0.927 to 0.975)	0.960 (0.940 to 0.981)	0.961 (0.942 to 0.979)	0.966 (0.948 to 0.983)	0.966 (0.949 to 0.982)
Stroke	0.968 (0.953 to 0.984)	0.967 (0.953 to 0.985)	0.975 (0.964 to 0.987)	0.976 (0.964 to 0.988)	0.979 (0.968 to 0.989)	0.979 (0.968 to 0.989)
Colorectal cancer	0.959 (0.938 to 0.979)	0.960 (0.940 to 0.980)	0.969 (0.953 to 0.984)	0.969 (0.954 to 0.984)	0.973 (0.959 to 0.986)	0.973 (0.959 to 0.986)
Incremental QALYs	3.6 (−10.7 to 17.9)		8.9 (−11.9 to 21.7)		4.4 (−12.6 to 21.5)	
Incremental costs	89 053 (12 328 to 164 492)		83 127 (2922 to 162 538)		83 767 (3219 to 162 945)	
Net Health Benefits	0.60 (−13.8 to 15.4)		1.93 (−15.5 to 19.4)		1.60 (−15.5 to 19.4)	
Probability cost-effective	52.9		57.3		55.9	

Figures represent the mean (95% interval) for 2000 simulations, except where indicated. QALYs costs and net health benefits are per 1000 participants.

## Discussion

### Main findings of this study

These results illustrate our approach to modelling the cost-effectiveness of interventions at different levels of deprivation. The results show that an effective intervention to promote physical activity has potential to give considerably greater benefits when targeted at people living in the greatest social and material deprivation, while a similarly effective intervention will yield fewer benefits in the least deprived groups. We showed previously^11^ that there is only weak evidence that such an intervention could be cost-effective overall. However, these results suggest that cost-effectiveness may differ across deprivation categories, with somewhat stronger evidence for cost-effectiveness in the most deprived category. It might be expected that an intervention to promote physical activity may be less effective, because of low uptake or attenuated effect, in lower socioeconomic groups. The present analyses suggest that an intervention that is 30% less effective in the most deprived category might have approximately similar cost-effectiveness to an intervention which retains its full effectiveness in the least deprived group. This observation points to the importance of considering the differential impact that health interventions might have in different strata of the population depending on socioeconomic status. This is clearly of crucial importance in developing public health interventions where intervention effectiveness may differ across these strata.^24^ Targeting interventions that may have greater potential cost-effectiveness in specific population strata may contribute to reducing health inequalities when health budgets are constrained.

### Limitations of this study

The study drew on data for a very large empirical sample of individuals registered in primary care to provide estimates of morbidity and mortality, as well as health-care utilization, for each single- and multi-morbid state included in the model. The model incorporated the major causes of morbidity associated with physical inactivity, as well as modelling multi-disease states, consistent with the frequent occurrence of multi-morbidity in primary care. Estimates for physical activity level by age, sex and deprivation category were drawn from a large national representative survey. Utility estimates were drawn from a compendium of previously reported values and these may not capture variations in the impact of disease across deprivation categories. Random error in the Model inputs was explicitly incorporated into the model in a probabilistic framework.

We acknowledge that it was necessary to make several assumptions. Existing intervention studies have evaluated outcomes up to 12, and in some cases 24 months. We assumed that a sustained intervention might have an effect on physical activity that lasted as long as the intervention. We also assumed that the time course of the effect would be the same in each deprivation category. We also assumed that the intervention could be delivered at very low cost, equivalent to the cost of one general practitioner consultation per year. Our previous research suggested that interventions delivered at higher cost than this are unlikely to be cost-effective,^11^ but we also performed sensitivity analyses on intervention cost. We acknowledge that interventions that are not effective will not be cost-effective so long as they are not less costly than standard treatment. However, the present results make a significant observation that any effective intervention has potential to be more cost-effective in lower socioeconomic groups. We also had to make assumptions about the degree to which the intervention might lose effectiveness in lower socioeconomic groups as there was no existing evidence guiding this estimate. For this reason we estimated a conservative range and evaluated interventions with higher costs for this sub-population as well. We only considered the use of physical activity interventions in primary prevention and we acknowledge that similar interventions might be used for secondary prevention after disease onset. The intervention was introduced into the model using a simplified measure of the frequency and duration of physical activity and did not incorporate measures of the intensity of physical activity because evidence on intervention effectiveness is not sufficient to support this at present. We included a limited range of disease conditions in the model, including major causes of morbidity associated with physical inactivity, but we acknowledge that there are other health outcomes that may be associated with physical activity. In spite of these limitations, we believe our results are important in drawing attention to the potentially greater cost-effectiveness of intervention at lower socioeconomic levels.

### What is already known on this topic

While the links between socioeconomic status and health are well established,^1^ their implications for economic evaluation are under-explored. To the authors' knowledge, previous studies have not systematically evaluated the cost-effectiveness of interventions in different strata of socioeconomic position or social and material deprivation.

### What this study adds

Socioeconomic status has a potentially important impact on estimated intervention cost-effectiveness for a primary care-based intervention to encourage physical activity. Even allowing that an intervention's effectiveness might be reduced in deprived circumstances, this study provides evidence that effective interventions to promote physical activity may have a greater likelihood of being cost-effective when implemented among individuals of lower socioeconomic status. These findings point to the importance of considering a stratified approach to evaluating the cost-effectiveness of public health interventions when need, in terms of capacity to benefit, varies systematically between population strata and budget constraints require allocation according to cost-effectiveness. The research also points to the importance of quantifying differences in intervention effectiveness between socioeconomic groups. We caution that even under favourable assumptions of cost and duration of effect, brief interventions in primary care appear unlikely to prove cost-effective as a universal strategy to promote physical activity.

## Funding

This study was supported by the UK National Prevention Research Initiative (http://www.npri.org.uk) whose funding partners include the Alzheimer's Research Trust; Alzheimer's Society; Biotechnology and Biological Sciences Research Council; British Heart Foundation; Cancer Research UK; Chief Scientist Office, Scottish Government Health Directorate; Department of Health; Diabetes UK; Economic and Social Research Council; Engineering and Physical Sciences Research Council; Health & Social Care Research & Development Office for Northern Ireland; Medical Research Council; The Stroke Association; Welsh Assembly Government; and World Cancer Research Fund. This research was supported by the National Institute for Health Research (NIHR) Biomedical Research Centre at Guy's and St Thomas' NHS Foundation Trust and King's College London. The views expressed are those of the author(s) and not necessarily those of the NHS, the NIHR or the Department of Health. Funding to pay the Open Access publication charges for this article was provided by the UK Medical Research Council.
